# From structures to systems: towards a model of ethical healthcare

**DOI:** 10.1080/20476965.2024.2436580

**Published:** 2024-12-07

**Authors:** Constantine Manolchev, Marco Campenni, Navonil Mustafee

**Affiliations:** a Sustainable Futures, University of Exeter Business School, Exeter, UK; b The Centre for Simulation, Analytics and Modelling (CSAM), University of Exeter Business School, Exeter, UK

**Keywords:** Ethical infrastructure, ethical system, bullying and harassment, complex adaptive systems

## Abstract

“Hurt people hurt people” is a phrase which summarises the cyclical nature of painful experiences and harmful actions. Arguably, this cycle of hurt and harm applies to the UK’s National Health Service (NHS), where employees are reporting record low levels of physical and mental wellbeing, while experiencing a climate of hostility, bullying and harassment, and pressures to meet targets. Such working environments carry several risks, not only for the employees themselves but also in terms of clinical outcomes for patients. As a result, a range of systemic and targeted infrastructure interventions have been trialled in several NHS hospitals (managed in the UK by independent Trusts), seeking to promote a culture of compassion, and improve the psychological safety of workers. However, the effectiveness of such measures in achieving ethical working environments and preventing unethical behaviours, has been questioned. We join the ongoing debate by proposing the need to go beyond ethical infrastructures and instead consider ethical environments in their systemic complexity. We conclude, by putting forward a model of a complex and ethical health system, which incorporates workplace networks, policy frameworks, and accounts for regional demographics.

## 1. From capability to “Perma-Crisis”

July 5 2023, marked the 75th anniversary of the UK’s National Health Service (NHS). Founded on the principle of providing free care at the point of delivery and employing approximately 1.2 million people, in 2021 it offered daily health services to an average of 1.3 million people in England alone (NHS Professionals, 2023). Yet, a King’s Fund report (Anandaciva, 2023) found that the NHS is “middle-of-the pack” when compared with the health systems in 18 peer countries in Europe, North America and Asia. Most notably, despite being able to secure service efficiencies, NHS staff have access to fewer resources and, overall, the NHS delivers poorer health outcomes for its patients (*ibid*.). Such findings are not altogether surprising. NHS staff report record lows of morale and wellbeing, with 40% suffering from work-related burnout (Best, 2021) and 30% considering leaving (NHS, 2021). Such experiences are anchored in an environment of organisational toxicity, which is experienced by staff across the NHS as a lack of compassion and psychological safety at work (Neal et al., 2023), evidenced by reduced ability and willingness to speak-up, share knowledge and ideas (O’donovan & Mcauliffe, 2020). This has led commentators to describe the NHS as permanently “broken” (Campbell, 2023) and even in a state of “perma-crisis”, with delays in accessing emergency care causing between 300 and 500 avoidable deaths per week (Badrinath, 2023).

In recognition of the risks to its patients, but also cognizant of the need to safeguard the health and wellbeing of its workforce, numerous NHS care units (or Trusts) have adopted interventions and measures to change their working environments. Notable among them are efforts to improve the psychological safety of working environments (Neal et al., 2023) and promote workplace compassionate, which – with support from compassionate leaders – is correlated with improved staff engagement and patient outcomes (West, 2021). Researchers have built on this foundation to suggest that compassion should be practised both towards others (colleagues and patients) and self (Clark et al., 2022). Manolchev et al. (2021) extend this further into an interlinked model of compassionate leadership, relationships and citizenship, embodying behaviours informed by self-care, camaraderie, and the building of personal resilience.

Workplace-focused approaches seem appropriate at first glance. The value of creating an ethical climate in organisations and the connection between (un)ethical climates and (un)ethical choices is recognised in a well-established body of literature, coalescing around several meta-reviews (Kish-Gephart et al., 2010). In this context, an ethical climate can be defined as individual perceptions and interpretations (S. R. Martin et al., 2014) of ethical organisational “practices and procedures”, which encourage and enable ethical behaviour (Victor & Cullen, 1988). Importantly the “ethical” construct encompasses both ethical and unethical behaviours (Treviño et al., 2014), as well as the shared interpretation of what is expected, what is sanctioned (Zohar & Luria, 2005) and what is rewarded in organisations (K. Einarsen et al., 2017). Ethical climates can contribute to perceptions of accountability and security, which can diminish abuse, aggression, and negative acts, such as bullying and harassment (K. Einarsen et al., 2017). In turn, both the ethical safety and ethical climate constructs have been associated with perceptions of psychological safety, organisational learning, creativity (S. R. Martin et al., 2014) and innovation (Schneider et al., 2013).

In a seminal contribution, Tenbrunsel et al. (2003) combine formal structures (such as policies and procedures), informal structures (personal relationships, custom and practice) with elements of the organisation’s climate, into an ethical infrastructure model. The model expects organisations to communicate with employees, provide organisational surveillance and issue sanctions in line with organisational attributes such as the corporate code of conduct, values, mission statements, training and so on. However, Tenbrunsel et al. (2003) also recognise the opaque relationship between ethical infrastructures and organisational outcomes whereby the former may, at times, reduce unethical behaviour but, at times, may not. This is so because formal and informal structures are nested in the context of the organisation’s culture, which exerts the strongest impact on (un)ethical behaviour (Ho et al., 2024). In this way, although formal systems may communicate organisational expectations of ethical behaviour through on-boarding, training, various codes of conducts and so on, informal systems may twist this message by allowing a raft of negative behaviours to take place (S. Einarsen et al., 2009). Thus, although Tenbrunsel et al. (2003) ethical infrastructure framework offers an important contribution to the literature, it does not – in its current format – present a model of the type of system deemed necessary to support employee wellbeing in the healthcare sector (Samuels et al., 2017), supporting recruitment in clinical and clerical roles (Camm et al., 2023), as well as delivering good patient care to the wider population (Kline, 2019).

## 2. Designing ethical systems

Developing a research agenda centred on designing health systems is well-aligned with the World Health Organisation’s healthcare strategy (Makleff et al., 2020; Swanson et al., 2012), as concretised in several flagship reports since 2000 (De Savigny & Adam, 2009; Van Olmen et al., 2012). Despite a clearly articulated endpoint, the task has significant challenges. Capturing real-life complexity in an (ethical) system model is fraught with conceptual and operational difficulties (Makleff et al., 2020). This is even more problematic when trying to account for ethical outcomes (Van Olmen et al., 2022), which is also the starting point for the Tenbrunsel et al. (2003) model. Finally, there is a lack of consensus on the boundaries of the system, which includes not only contexts, providers, and users of care but also the wider social, political, and economic environment (Sheikh et al., 2014).

The design of health systems thus presents itself as a wicked problem (Rittel and Webber, 1973), with scholarly contributions at times exacerbating rather than alleviating existing and entrenched inequalities in the sector (Porroche-Escudero, 2022). Nevertheless, we argue that it is necessary to replace static ethical typologies with system thinking strategies, acknowledging dynamic interactions within the organisation, and information exchanges with its environment. Furthermore, system thinking strategies can lead to ongoing, iterative learning, which is particularly important for health systems which are complex adaptive systems (CAS) that demonstrate the characteristics of constant evolution, self-organisation, resilience, non-linearity, and feedback loops (Swanson et al., 2012). This approach can be extended to recognise the relationship between the health systems and the wider population. This can be observed both in the context of positive healthcare outcomes (delivered by the health system to the wider public) and human resources (recruited by the health system from the wider public) (see, for example, Kline & Lewis, 2019).

Guided by systems thinking principles (Adam & de Savigny, 2012) we identify four key elements in this paradigm shift from structures to systems. First, a shift from formal and informal structures (Tenbrunsel et al., 2003) which tend to be static by nature, towards *dynamic relationships* (Sheikh et al., 2014; Van Olmen et al., 2012) acknowledging the dynamic nature of roles within the organizations, as well as the interpersonal dynamics influenced by emotions, values, and culture. Such a shift can account for the non-linearity of complex systems and time-lags between implementation and visible outcomes (Swanson et al., 2012). Second, a shift from hierarchy of system structures, which contains different levels of ranking in terms of impact and significance, towards *decentralised networks* (*ibid*.), i.e., social network configurations where there are multiple key nodes serving as a hubs for participants (e.g., different authorities). Third, a shift from “factor-thinking”, which considers individual conditions (factors) that may correlate with results and towards *loop-thinking*; the latter incorporates iterative feedback loops and reflects system changes over time (Adam & de Savigny, 2012). Thus, unlike factor-thinking which assumes a one-way directional relationship, loop-thinking accepts that relationships among factors can be an ongoing process and not a one-time event, with effect(s) providing feedback to affect the causes and the causes affecting each other. Fourth, loop-thinking is *place-based*, that is, anchored in local contexts, reflecting the population impact of healthcare on a particular region (Mortimer et al., 2018). Place-based approaches are usually collaborative, long-term approaches aiming at building thriving communities delivered in a defined geographic location, recognising the importance of addressing the wider determinants of health (the conditions into which people are born, live and work) across the life course. Thus, instead of focusing on individual conditions at a single stage in life, the focus should be on critical stages, transitions and settings.

## 3. The Place-based Ethical Environment System (PACEM) Model

**Figure 1.**
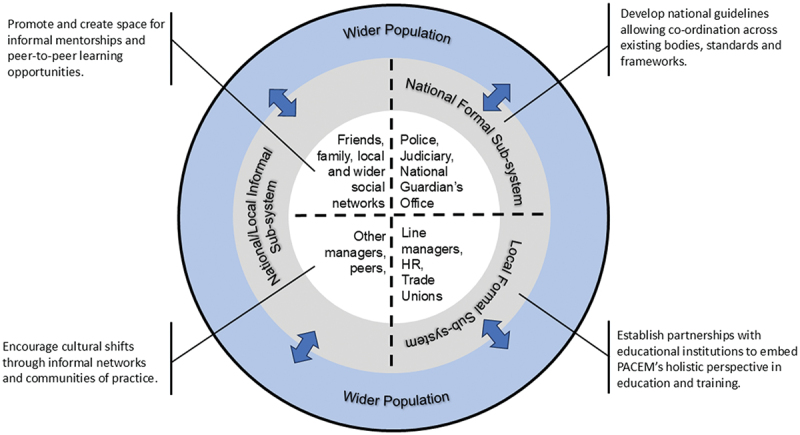
PACEM system dimensions and strategic intersections.

We operationalise those four principles into formulating the conceptualisation of a place-based ethical environment system (PACEM - ***p***lace-b***a***sed ethi***c***al ***e***nvironment syste***m***), naming the acronym after the Latin term for “peace” (See Figure 1 below).

The PACEM Model (Figure 1) seeks to represent complex relationships between multiple actors, operating across different levels and within different timescales. It has three levels – component, sub-system and population. There are three sub-systems, a national formal sub-system, a local formal sub-system, and distinct but overlapping national/local informal sub-system. Although each sub-system has its distinct components, there is close interaction across all three, and we have recognised this with the dotted lines in Figure 1, which identifies the four quadrants of the PACEM model. The four lines linking the outermost concentric circle with the centre indicate overlap across levels, namely, between local/national sub-systems and the wider population. We describe each in more detail below.

The national formal sub-system includes, as its components, a complex network of organisations that are external to the NHS. Examples include, the Police, the Judiciary (Courts of Law), and, in the UK, whistleblowing bodies such as the National Guardian’s Office that govern the institutional context of healthcare (Currie & Finnegan, 2011). This sub-system recognises the accountability of healthcare organisations, which are subject to both regulation and investigation (D. Hughes et al., 2011). Operationalising the PACEM model at a national, formal sub-system level can allow it to align existing national healthcare policies and regulations in the context of ethical standards and guidelines. It can also enable compliance and whistleblowing mechanisms to be standardised and thus normalised across different NHS Trusts.

The local formal sub-system incorporates members of the organisation’s hierarchy, including managers, human resource (HR) professionals and trade union representatives. Those components have a critical role in tackling bullying and harassment behaviours (Lewis and Kline, 2019) and also ensuring high standards of ethical behaviour. This creates an opportunity to use training and education as a means of supporting the design of ethical healthcare systems. Here, NHS Trusts can establish partnerships and co-design training programmes allowing managers to upskill and better manage workforce workload and organise employee relations (Corby, 2000). Bespoke learning provisions can be tailored to the needs of Trade Unions representatives, Freedom to Speak-Up Guardians and HR personnel (H. Hughes, 2019), offering personal development opportunities and allowing each function to continuously adapt to Trust-specific pressures and support local employees and patients. Those measures can be further supported by measuring and evaluating impact and performance through existing mixed methods such as surveys and performance reviews, as well as commissioning academic researchers to run focus groups, carry-out interviews, and so on.

The informal sub-system consists of both formal and informal relationships in organisations, which may expand across local and national networks and can include peer and bystander relationships (Ng et al., 2020). As such, the sub-system’s components include interactions in everyday settings and contexts. For example, these may be peer-to-peer conversations to share information, to vent frustrations or to share rumours. It may be discussions between employee and experienced members of staff that do not directly line-manage the employee. The interactions could include knowledge sharing across NHS communities of practice and wider personal networks (Amery & Griffin, 2020). Operationalising the PACEM model at this sub-system level can be achieved by allowing staff space to seek informal mentorship, or engage in peer-to-peer learning, for instance, by cultivating their own community of practice and becoming individual drivers for a shift towards a more ethical and compassionate culture (Manolchev et al., 2021).

Lastly, the PACEM model has a place-based boundary and accounts for the demographics of its wider, regional population. This recognises the need to deliver positive healthcare outcomes to patients but also put in place prevention strategies (Khan et al., 2023). It also promotes the importance of staff-patient relationships, whereby positive health outcomes can be promoted through patient empowerment (Nygårdh et al., 2012) and the building of trust (Brennan et al., 2013). Healthcare is socially embedded (D. Hughes et al., 2011) across personal and professional connections, internal and external social networks (Webber et al., 2015), and thus creating psychological safety and ethical working environments are critically important to recruit and manage the human resources of the health system.

In defining and designing the conceptual PACEM model, we envisage that it could be formally operationalised and implemented following the principles and the concepts of complex adaptive social systems (Preiser et al., 2018). The PACEM model is characterised by multi-level, multi-actor, and different temporal dynamics. Thus, we suggest that among the different modelling techniques available, a hybrid modelling approach combining methods and research approaches from disciplines such as computer modelling and simulation, social sciences, and management may be considered for transdisciplinary research (Tolk et al., 2021). For example, in a separate study on bullying and harassment in healthcare organisations (Ho et al., 2024), the authors developed a agent-based social simulation model that combines the following three approaches: i) agent-based modelling and social simulation (Smaldino, 2023), ii) social network theory and analysis (Borgatti & Ofem, 2010), and iii) use of empirical data (to parameterise the agent-based model). Such a hybrid approach to model an ethical healthcare system would allow us to define the extant behaviours of different actors that are identified in the four quadrants of the PACEM model, e.g., friends, family, managers, peers, HR and trade unions, their relationships, and to simulate adaptive dynamics resulting from the actors’ (inter)actions within a specific social context.

## 4. Conclusions

Safeguarding the health and wellbeing of society is a crucial task for Government, and the NHS is the cornerstone of healthcare in the UK. It is however, faced with multiple pressures – growing waiting lists for elective procedures (Iacobucci, 2017), longer wait time for urgent and emergency care (Mustafee et al., 2024) and aspirational performance targets (Health Foundation, 2017), historic difficulties with resource allocation (The Marmot Review, 2010) and digital transformation challenges (NHS England, 2023). To successfully address such complex challenges, the NHS needs to be able to not only recruit but also retain employees in clinical and administrative roles. In the above opinion piece, we have argued that this is predicated on creating an ethical system, able to protect the psychological safety and emotional resilience of healthcare employees.

Our PACEM model proposes such an ethical system. We recognise that NHS Trusts are complex systems, connected to national and local networks, operating in specific regional contexts with social, demographic and geographic implications. Such considerations determine not only the experience of employees within each Trust, but also the quality and accessibility of care for the population in a specific region. By outlining the national formal, the local formal, and thenational/local informal sub-systems supporting the provision of ethical healthcare, and considering the wider population of the ethical system, we put forward a new research agenda. We call for the study of the healthcare sector in its holistic and systemic complexity (see Campenni et al., 2022 for an example of such an approach). In this opinion piece we offer a way forward and show how the sub-systems we identify can be connected and utilised towards ethical outcomes. More work is needed to show how such integrations can be evaluated and embedded in individual NHS Trust policy.
